# Clinico-epidemiological profile and treatment outcomes in patients with squamous cell carcinoma of the esophagus following docetaxel-based neoadjuvant chemotherapy: experience from a cancer care center in Northeast India

**DOI:** 10.1186/s43046-021-00094-0

**Published:** 2021-10-21

**Authors:** Partha Sarathi Roy, Gaurav Kumar, Sreya Mallik, Satya Sadhan Sarangi, Bhargab Jyoti Saikia, Munlima Hazarika, Abhijit Talukdar

**Affiliations:** 1 Department of Medical Oncology, Dr. B Borooah Cancer Institute, Guwahati, Assam India; 2 Department of Surgical Oncology, Dr. B Borooah Cancer Institute, Guwahati, Assam India

**Keywords:** Esophageal cancer, Squamous cell carcinoma, Neoadjuvant Chemotherapy, Pathological complete response

## Abstract

**Background:**

Squamous cell carcinoma of the esophagus ranks as the most common cause of cancer incidence and mortality in males and the second most common in females. Surgery alone is associated with poor long-term survival. Neoadjuvant chemoradiation and perioperative chemotherapy without radiation have been tried to improve survival rates.

**Methods:**

We retrospectively evaluated the neoadjuvant chemotherapy in forty-eight patients with non-metastatic, non-cervical squamous cell carcinoma of the esophagus with a docetaxel-based three-drug regimen to improve complete pathological response rates.

**Results:**

The median age of presentation was 52 years, with male preponderance. All the patients received three cycles of docetaxel-cisplatin-fluorouracil-based chemotherapy. A complete pathological response to neoadjuvant chemotherapy was seen in 8 patients (17%). Rates of grade 3 hematological toxicities were seen in 12% of patients, with no observed grade 4 toxicity. The most common non-hematological toxicity was grade 3 alopecia (seen in 40%) and grade 2 nausea/vomiting in 8% of patients. At a median follow-up of 26.5 months, 2-year survival for the patients receiving chemotherapy and surgery is 66%.

**Conclusions:**

Preoperative chemotherapy with a taxane-based triple-drug regimen is a reasonable approach in squamous cell carcinoma of the esophagus, associated with improvement in complete pathological response rates, increases complete resection rates, with manageable toxicity.

## Background

Squamous cell carcinoma of the esophagus (SCEC) is the 8th most common cancer diagnosed globally and 6th most common in terms of cancer-related mortality [1]. Both the burden of the disease and the tumor-related mortality are even higher in Northeast India. It ranks as the most common cause of cancer incidence and mortality in males and the 2nd most common in females [2]. Due to the lack of endoscopic screening programs, most patients present in locally advanced stages, ultimately requiring multi-modality treatment. Recent surgical series results report 5-year survival rates of 15 to 20% for surgery alone [3–7]. Therefore, additional treatment modalities are needed to improve the outcomes. One such option is the usage of neoadjuvant concurrent chemoradiation (NACCRT). NACCRT had shown to increase survival rates compared to the surgery-alone arm in some randomized studies, albeit with a significantly increased rate of perioperative complications [8, 9]. An alternative approach to this strategy is perioperative chemotherapy without radiation, a more preferred option in Japan. In a Japanese Study ( JCOG 9907) which compared neo-adjuvant chemotherapy (NACT) with postoperative chemotherapy in the treatment of stage II/III esophageal squamous cell carcinoma, preoperative chemotherapy with cisplatin and 5-fluorouracil (5-FU) followed by surgery was shown to improve OS (5-year survival—55%) without additional serious adverse events [10]. But to further increase the response and survival rates, various authors have tried to add taxanes to the backbone of cisplatin and 5-fluorouracil-based chemotherapy. Studies in advanced gastric carcinoma and squamous cell carcinoma of the head and neck region have already shown improved response and survival rates to triplet docetaxel, cisplatin, and 5-fluorouracil (DCF)-based NACT [11–13]. Chan’s meta-analysis showed that complete pathological response (pCR) to NACCRT is higher than NACT [14]. It has also been demonstrated in various randomized trials that higher pCR rates translate into a survival benefit [15, 16]. In this analysis, the NACT regimen comprised of two drugs (cisplatin and 5-fluorouracil). But when taxane is added to this doublet, the pCR might increase. But not many studies, especially from the Indian subcontinent, have analyzed the pCR rates of DCF-based NACT in the esophagus’s squamous cell carcinoma (SCEC). Our present study sought to investigate the pCR rates to DCF-based NACT in patients with SCEC.

## Methods

### Study design

This was a retrospective observational study to evaluate the pCR rates and median survival in patients of non-metastatic squamous cell carcinoma of the esophagus (SCEC), who had received DCF-based NACT and underwent surgery at our institute between January 2016 and December 2017. The study received the institutional ethics committee (IEC) approval.

### Participants

Out of a total of 2450 cases of esophageal cancers diagnosed from January 2016 to December 2017 at our center, clinical data was obtained from 48 diagnosed patients of primary SCEC. The inclusion criteria included are (1) biopsy-proven squamous cell cancer of the esophagus, (2) age ≥ 18 years, (3) both sexes, (4) stage III disease, and (5) information available regarding demography, clinical characteristics, and treatment parameters. Exclusion criteria included (1) squamous cell carcinoma of the cervical esophagus, (2) age less than 18 years, (3) patients with other histologies like adenocarcinoma and small cell carcinoma, (4) patients who underwent preoperative chemoradiation or radiation only, (5) stage I, II, and stage IV, (6) patients who underwent surgery upfront, (7) patients who did not undergo surgery after DCF-based NACT, and (8) patients who received NACT regimen other than DCF.

Data collection and follow-up notes were collected retrospectively from hospital-based cancer registries; individual medical case notes; electronic patient records; and pathology reports, including age, gender, performance status, history of smoking, tobacco and alcohol intake, history of any medical risk factors, symptom burden, the grade of dysphagia, size of the primary tumor, histological type, stage, site, and socioeconomic background. A detailed retrospective chart review was performed to document staging, endoscopic findings, therapy, follow-up, and survival outcome. The staging was done using American Joint Committee on Cancer Staging System (AJCC), the 7th edition. All patients received an endoscopic biopsy before treatment. Staging workup included a physical examination, chest radiography, and CT scan of the abdomen. Patients received surgery, chemotherapy, and radiotherapy, either alone or in combinations. Patients diagnosed with stage III SCEC were taken up for three cycles of 3 weekly docetaxel at 75 mg/m^2^ day 1, cisplatin at 75 mg/m^2^ day 1, and 5-fluorouracil at 750 mg/m^2^ day 1‑day 5-based NACT along with prophylactic growth factors support. This was followed by surgery in responsive patients. Surgery was carried out within 42 days of the completion of chemotherapy. Patients underwent right or left thoracotomy for curative resection by total or subtotal thoracic esophagectomy or trans-Hiatal esophagectomy. A laparoscopic procedure for esophagectomy was permitted. Regional lymphadenectomy consisted of two- or three-field extended lymphadenectomy.

Evaluations of residual tumor (R) were classified as follows: R0, no residual tumor; R1, suspicious of residual tumor or microscopic residual tumor; or R2, macroscopic residual tumor. Surgical specimens were evaluated pathologically for the assessment of complete response in histopathology specimens. If no cancer was seen, i.e., ypT0N0Mx, this is considered a pathologically complete response or considered a residual disease.

### Statistical methods

Patient and demographic features were summarized using median/centiles, means, and standard deviations (SD). Treatment outcomes were presented in percentages. Survival was analyzed with the Kaplan-Meier method. Analyses were performed in the SPSS 16.0 software.

## Results

### Demography (Table 1)

**Fig. 1:**
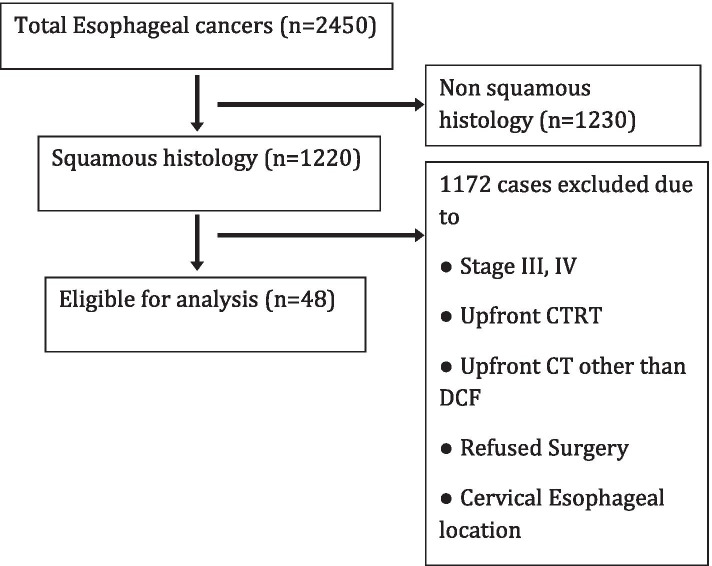
Consort diagram

Of the total 2450 cases of esophageal cancers, 48 patients with SCEC were eligible for the study analysis (Fig. 1). The median age of presentation was 52 years (range 39‑65) with male preponderance (male to female ratio was 2:1). All the patients have an ECOG performance status of 1. The median distance that the patients had to travel to reach the hospital was 160 km, ranging from 5‑550 km. Two-third (32/48) of these patients belonged to the rural locality. Thirty-seven (37%) percent of these patients were farmers by profession. More than two-thirds (70%) of these patients were smokers. History of alcohol intake was present in 45 % of patients. None of these patients had any family history of cancer.

**Table 1:** Demographic parameters

Characteristics (***N*** = 48)	Frequency (%)
**Distance from the native place (in km)**	
Mean ± SD, range	200 ±172, 5‑550
Median, Q1‑Q3	160, 52‑300
**Age in years**	
Mean ± SD, range	51 ± 8; 39‑65
Median, Q1‑Q3	52; 43‑59
**Gender**	
Male	32 (66)
Female	16 (34)
**Median ECOG**	1
**Locality**	
Rural	32 (66)
Urban	16 (34)
**Occupation**	
Farmers	18 (37)
Housewife	16 (34)
Businessman	10 (20)
Others	4 (9)
**Smokers**	
Yes	34 (70)
No	14 (30)
**Alcohol intake**	
Yes	22 (45)
No	26 (55)

### Disease characteristics (Table 2)

Majority of the patients presented with bulky tumors. The median size of the primary tumor was 6 cm (range 4‑11 cm). The most common site of involvement was the middle-third esophagus, followed by the lower-third and upper-third. Forty-two out of 48 patients (87%) had radiologically node-positive disease. Moderately differentiated squamous cell carcinoma was the most common histology in 70% (34/48) of patients. Dysphagia was the most common symptom, and the majority of them presented with grade II dysphagia seen in 66% of patients.

**Table 2:** Disease characteristics

Characteristics (***N*** = 48)	Frequency (%)
**Dysphagia**	
Grade 0	0 (0)
Grade 1	4 (8)
Grade 2	32 (67)
Grade 3	12 (25)
Grade 4	0 (0)
**Site**	
Upper third	6 (12)
Middle third	26 (54)
Lower third	16 (34)
**Nodal involvement**	
Yes	42 (88)
No	6 (12)
**Tumor size (in cm)**	
Mean ± SD, range; median, Q1‑Q3	6.4 ± 1.6, 4‑11; 6, 5‑8
**Grade**	
Well-differentiated	6 (12)
Mod-differentiated	34 (70)
Poor-differentiated	8 (18)
**Weight loss**	
Yes	44 (92)
No	4 (8)

### Treatment parameters and response (Table 3)

**Fig. 2:**
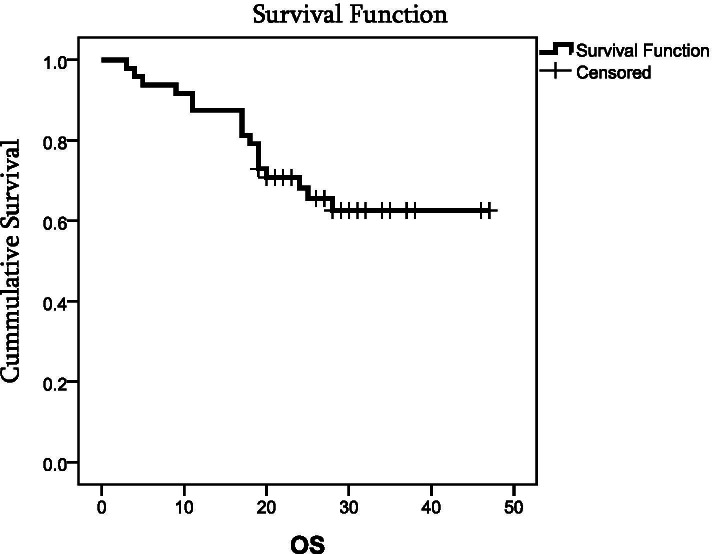
Kaplan-Meir curve showing overall survival (in months)

Of the 48 patients, all the patients received the planned three cycles of DCF-based NACT followed by surgery. No dose reductions were required in any cycles. However, there were delays between cycles due to grade 2 neutropenia and grade 3 anemia (14/48; 30%) despite growth factor support. The median time to completion of NACT was 52 days (range 43‑68). Twelve percent (12%) experienced grade 3 anemia, and grade 2 neutropenia occurred in 58% of the patients. There were no grade 4 toxicities during the NACT phase. The median gap between the last cycle of NACT and surgery was 31 days (range 22‑44). The pCR rates to DCF based NACT was 17% (8/48). All patients who underwent surgery received extended lymphadenectomy, and 42 of 48 patients underwent R0 resection pathologically. Also, there was no intraoperative or immediate postoperative mortality. At a median follow-up of 26.5 months, 2-year survival for the patients receiving chemotherapy and surgery is 66% (Fig. 2).

**Table 3:** Treatment parameters and response

Characteristics (***N*** = 48)	Frequency (%)
**Median no. of chemotherapy cycles**	3
**Most common toxicities**	
Grade 2 neutropenia	28 (58)
Grade 3 anemia	6 (12)
Grade 2 vomiting	4 (8)
None	10 (22)
**Median treatment time for NACT completion (range) in days**	52 (43‑68)
**Median time gap between the end of NACT and surgery (range) in days**	31 (22‑44)
**Treatment response**	
Pathological CR (pCR)	8 (17)
Residual disease (less than pCR)	40 (83)
**R0 resection rates**	
Yes	42 (88)
No	6 (12)
**2-year survival (%)**	66

## Discussion

Despite being a retrospective analysis, this study provides evidence regarding favorable outcomes with an intensification of preoperative chemotherapy by adding taxane to cisplatin and 5-fluorouracil backbone. Compared to doublet chemotherapy, the triple-drug regimen augments the pCR rates, and in turn, can lead to better outcomes in locally advanced esophageal cancers. Clinico-epidemiological characteristics when compared to other studies on esophageal cancer, our study found a lower median age at presentation, 52 years in the present study compared to 60‑62 years in different classes [17]. The proportion of female patients was more (34%) in our study, probably reflecting the higher prevalence of tobacco consumption in females around this part of the country [18]. Two-thirds of patients in this study belonged to a rural background and were farmers by profession. There is a high prevalence of tobacco smoking (70%) and chronic alcohol consumption (45%) in patients included in our study compared to other Indian and international studies [18, 19]. Dysphagia was the most common presenting symptom, with more than two-thirds of patients with grade 2 or higher dysphagia. The commonest location was the middle third in 54% of patients, followed by the lower third in 34%. According to the site, this data does not provide the actual distribution estimates, as patients were selected retrospectively based on surgical resectability. In our study, 88% of patients had radiologically node-positive disease. The median length of wall involvement was 6 cm, which is higher than 4 cm found in the CROSS trial but was similar to other studies reported from other low-income countries [20, 21]. Significant weight loss (> 10% over the last 6 months) was reported in 92% of patients in this study, which was way higher than 25% found in a survey by Boonstra et al. [22]. This study found patients in this region of India have the more advanced and bulky disease, along with a very high number of participants with significant weight loss, thus, making the multidisciplinary approach most suitable and challenging in this group of patients.

Treatment compliance and outcomes with chemotherapy were found to be good in our study. All the participants completed planned three cycles of preoperative chemotherapy with growth factor support used as primary prophylaxis in our study compared to the compliance rate of 88% reported by Boonstra et al. [22]. The rate of grade 3 hematological toxicities were 12%, and there was no grade 4 toxicity in this study of triplet chemotherapy as compared to 10% grade 4 hematological study reported by Boonstra et al. with doublet chemotherapy and 25% grade 3‑4 toxicity reported by MAGIC trial with triplet anthracycline-based chemotherapy [22, 23].

Non-hematological toxicity in the form of grade 3 or more alopecia was found in 40% of patients. The rest of the non-hematological toxicity was grade 2 nausea and vomiting in 8% of participants. There were no excessive untoward delays found in either completing three chemotherapy cycles or performing surgery after chemotherapy. The median duration to complete three cycles of preoperative chemotherapy was 52 days, and the median gap between completion of chemotherapy to the date of the surgery was 31 days, which was similar to that reported in the MAGIC trial (99 days from randomization to surgery) and 97 days reported by a phase III Swedish trial [23, 24]. In our study, 88% of patients underwent R0 resection, which was higher than compared to other studies on preoperative chemotherapy such as one by Medical Research Council Esophageal Cancer Working Group, which reported 60% R0 resection rates and 62% R0 resection by Kelsen et al. [3, 25]. As compared to trials using preoperative chemoradiotherapy our study has lowered R0 resection rates, i.e., 88% versus 92‑100% in preoperative chemoradiotherapy studies [17, 20]. Pathological complete response rates to NACT in this study was found in 8 patients (17%) as compared to 13% in the study by Ancona et al., 9% by Swedish phase III trial, 7% by Boonstra et al., and 2.5% reported by Kelsen et al. [3, 15, 22, 24]. This study clearly showed higher pCR rates with the addition of taxane to cisplatin and fluorouracil backbone. At a median follow-up of 26.5 months, 2-year survival in our study was 66%, higher than 42% reported in the study by Boonstra et al. compared to 80% estimated 2-year survival found in a study from Japan [17, 22]. Despite a good response to triplet chemotherapy in our study, pCR rates in this study are lower than those attained by preoperative CRT (ranges from 39‑49%) [20, 26].

## Limitations

This study’s retrospective nature precludes us from arriving at a definitive conclusion regarding the role of preoperative chemotherapy in carcinoma esophagus. Small sample size and shorter follow-up are other significant limitations of our study.

## Strength

Studies on preoperative chemotherapy from this part of the country are limited. Moreover, studies using triplet taxane-based preoperative chemotherapy in the era of preoperative CRT are also fewer from India. Our study is one of India’s very few studies reporting pCR rates of triplet taxane-based preoperative chemotherapy.

## Conclusions

This study showed that response rates by the addition of taxane to cisplatin and 5-fluorouracil-based backbone improves pCR and also R0 resection rates in carcinoma esophagus. The toxicity profile of taxane-based preoperative chemotherapy is also manageable and comparable to doublet chemotherapy with the prophylactic use of growth factors. Despite triplet preoperative chemotherapy, response rates achieved with this combination are inferior to those achieved with preoperative CRT. Prospective trials are needed to validate the results and compare preoperative triplet chemotherapy with preoperative CRT in the esophagus’s squamous cell carcinoma.

## Data Availability

Obtained from hospital-based case records and hospital information services.

## Abbreviations

SCEC: Squamous cell esophageal carcinoma
NACCRT: Neoadjuvant concurrent chemoradiation
NACT: Neo-adjuvant chemotherapy
OS: Overall survival
5FU: 5 Fluorouracil
pCR: Pathological complete response
DCF: Docetaxel-cisplatin-5 fluorouracil
IEC: Institutional ethics committee
AJCC: American Joint Committee on Cancer Staging System
SD: Standard deviations
R: Residual tumor
R0: No residual tumor
R1: Microscopic residual tumor
R2: Macroscopic residual tumor
CRT: Concurrent chemoradiation
